# Feasibility of an Isolated Kidney Perfusion Model for Postmortem Interval Estimation in a Rabbit Model: A Pilot Study

**DOI:** 10.3390/diagnostics16091266

**Published:** 2026-04-23

**Authors:** Ramazan Temürkol, Hülya Güler, Ahsen Kaya, Orhan Fahri Demir, Meltem Kocamanoğlu, Yasemin Akçay, Ayşegül Keser

**Affiliations:** 1 Department of Forensic Medicine, Faculty of Medicine, Ege University, Izmir 35100, Turkey; ramazantemurkol@gmail.com (R.T.); ahsen.kaya@ege.edu.tr (A.K.); 2 Department of Plastic, Reconstructive and Aesthetic Surgery, Faculty of Medicine, Ege University, Izmir 35100, Turkey; orhanfahridemir@hotmail.com; 3 Department of Medical Biochemistry, Faculty of Medicine, Ege University, Izmir 35100, Turkey; meltemkcmnoglu@gmail.com (M.K.); yasemin.akcay@ege.edu.tr (Y.A.); 4 Department of Physiology, Faculty of Medicine, Ege University, Izmir 35100, Turkey; aysegul.keser@ege.edu.tr

**Keywords:** postmortem interval, isolated kidney perfusion, supravital reaction, forensic medicine, feasibility study, postmortem changes

## Abstract

**Background:** The estimation of the postmortem interval (PMI) remains a complex challenge in forensic medicine. While macroscopic, biochemical, and molecular methods are well-documented, postmortem functional approaches at the organ level are largely underexplored. This pilot study investigated the feasibility of utilizing an isolated ex vivo kidney perfusion model to assess residual postmortem renal function—specifically glomerular filtration and tubular solute handling—as a potential chronological marker for PMI. **Methods:** Sixteen adult New Zealand rabbits were euthanized and randomly assigned to four postmortem interval groups (1, 5, 10, and 15 h). An unoxygenated, room-temperature crystalloid perfusion system was established to mimic natural postmortem decay. Initially, 32 kidneys were perfused; two were excluded due to anuria, resulting in 30 successfully analyzed kidneys. To strictly eliminate pseudoreplication bias, bilateral functional data were mathematically aggregated at the subject level, establishing the individual rabbit (*n* = 16) as the statistical unit. **Results:** Following statistical adjustment at the subject level, none of the measured functional parameters exhibited statistically significant chronological variation across the postmortem intervals (all *p* > 0.05; statistical significance defined as *p* < 0.05). Glomerular filtration was profoundly depressed across all groups, with adjusted inulin clearance ranging between 0.0031 and 0.0086 mL/min/g (peaking nonsignificantly at 10 h). Furthermore, active tubular reabsorption was virtually nonexistent; calculated reabsorbed loads for evaluated solutes, particularly potassium and sodium, yielded predominantly negative values. This phenomenon indicates a complete absence of physiological active reabsorption, reflecting instead a massive passive leakage of intracellular electrolytes into the tubular fluid due to cellular autolysis. **Conclusions:** Within this specific experimental setup, the isolated kidney perfusion model failed to demonstrate reproducible, time-dependent renal function useful for PMI estimation. These findings indirectly suggest that, unlike the prolonged supravital physiological resilience observed in skeletal muscle, highly metabolically active renal tissue rapidly loses its complex functional capacity following somatic death. Future studies exploring supravital renal function should consider targeting the immediate early postmortem period (0–1 h) or integrating advanced organ preservation techniques to unmask residual cellular capabilities.

## 1. Introduction

As a result of the disruption of vital functions, the delivery of blood and oxygen to tissues and organs ceases. Depending on whether this ischemia/anoxia condition is reversible or not, the process results in “survival” or “death.” In reversible ischemia, latency, survival, resuscitation, and recovery phases occur sequentially [1,2]. In cases where ischemia is irreversible, death occurs. The irreversible cessation of blood and oxygen delivery to tissues and organs represents the beginning of a period during which tissues can survive under ischemic conditions; this is referred to as the “supravital period” or “intermediate life” [1].

The difference between the resuscitation period and the supravital period is defined by the “reversibility/irreversibility” of the damage. Studies in the fields of physiology and experimental surgery have demonstrated that the duration of the supravital period is much longer than that of the resuscitation period [3]. For instance, while the resuscitation period in skeletal muscle is approximately 2–3 h, it has been shown that the supravital reactions of muscles, i.e., their electrical excitability, can extend up to 20 h postmortem (PM) [1,3]. The basis of supravital reactions is the continuation of metabolism until substrates are depleted or reaction-limiting changes in the cell occur after death [1].

The estimation of the postmortem interval (PMI) remains a complex and pivotal challenge in Forensic Medicine [4,5]. In routine practice, PMI determination relies heavily on the assessment of early postmortem changes—such as algor mortis, livor mortis, and rigor mortis—which often suffer from wide confidence intervals due to environmental dependencies [6]. For a PMI estimation method to be practically useful in routine forensic investigations, it should ideally provide a temporal resolution with a narrow margin of error (e.g., within a window of a few hours). While recent research has largely shifted toward the cellular and molecular levels [4], and existing methods introduced for examining physiological processes focus heavily on localized cellular or tissue-level functions, there is a distinct lack of studies investigating postmortem functional capacity at the intact, whole-organ level [1,2,7]. Studies investigating functions at the organ level are mostly related to organ transplantation, pharmacology, and physiology, focusing on recovery rather than natural postmortem degradation [1,2,7].

To bridge this knowledge gap, the kidney offers unique anatomical and physiological advantages over other organs. Unlike skeletal muscle, the kidney’s architecture allows for the precise, ex vivo quantification of complex metabolic and filtration tasks—such as the glomerular filtration rate (GFR) and specific solute handling (e.g., reabsorption and secretion in different nephron segments)—through established isolated perfusion models [8,9]. Furthermore, compared to the lower basal energy demands and relative hypoxia tolerance of muscle tissue [10], the highly metabolically active kidney is profoundly vulnerable to ischemia [11,12], making its postmortem functional degradation potentially more sensitive to chronological tracking.

Crucially, postmortem ischemic damage in the kidney is not structurally homogeneous, which provides a unique opportunity to track time-dependent changes. Previous histopathological and enzyme histochemical studies have demonstrated a chronological progression of differential segmental autolysis [13,14]. Due to high baseline Na,K-ATPase activity and rapid ATP depletion, the distal tubules and the thick ascending limb of Henle’s loop undergo severe autolysis as early as 5 to 10 h postmortem [13]. In contrast, the proximal tubules exhibit greater morphological resilience, with severe autolytic changes, such as cytoplasmic vacuolization, only becoming prominent around 15 h postmortem [13]. Furthermore, critical structural deterioration and significant shifts in oxidative stress markers (e.g., MDA and NO) in renal tissue have been shown to initiate around the 5- to 6 h mark [14,15].

Therefore, this pilot study aimed to explore the feasibility of isolated kidney perfusion as a functional model in the postmortem period. Rather than assessing the immediate phase of reversible warm ischemia (0–1 h), the experimental postmortem intervals evaluated in this study (1, 5, 10, and 15 h) were specifically selected to correspond with these established chronological milestones of segmental autolysis. The primary hypothesis of our study explores whether this sequential, segment-by-segment degradation of the nephron during the supravital period could be conceptually detected functionally through measurable alterations in specific solute handling and clearance under an experimental perfusion model. The secondary hypothesis was to explore whether such a renal functional model may have potential in PMI estimation, serving as a basis for future preliminary exploration.

## 2. Materials and Methods

### 2.1. Experimental Animals

This study used 16 adult New Zealand rabbits (eight male, eight female) weighing between 1330 and 3210 g. Although this weight range is relatively broad, all subsequent functional parameters (e.g., flow rate and clearances) were mathematically adjusted per gram of kidney weight to eliminate any size-dependent bias. The rabbits were young adults aged between 4 and 8 months. The experimental animals were obtained from the Saki Yenilli Experimental Animals Production and Application Laboratory. To ensure baseline health the animals were maintained under standard laboratory conditions at the Ege University Laboratory Animals Application and Research Center. The animals were housed in a temperature-controlled environment under a 12 h light/dark cycle, with ad libitum access to standard pellet chow and fresh water. No fasting protocols were applied prior to the experiments. They were transferred to the experimental laboratory solely on the day of the procedure to minimize pre-procedural stress, in accordance with the ARRIVE guidelines [16] (Supplementary File S1).

### 2.2. Experimental Groups and Euthanasia

The subjects were randomly allocated to the postmortem interval study groups (PM-1, PM-5, PM-10, and PM-15) by drawing lots (simple randomization), with each group containing two females and two males. There were no statistically significant differences regarding age or baseline body weight among the groups (*p* > 0.05). Following randomization, the animals were euthanized using high-dose CO_2_ at time points corresponding to the planned postmortem intervals. High-dose CO_2_ euthanasia was selected to ensure rapid CNS inhibition and minimize variable pre-mortem hypoxic stress. While CO_2_ may induce acute changes such as respiratory acidosis and potassium shifts [17], these are vastly overshadowed by the autolytic degradation and global tissue hypoxia occurring during the advanced postmortem intervals evaluated (1–15 h). This protocol guaranteed an identical biochemical baseline at time zero, effectively eliminating the mode of death as a confounding variable. The euthanized rabbits were kept intact at room temperature (24 ± 1 °C) under standard ambient lighting and a relative humidity of ~40–50%, without active airflow or temperature control, until surgical procedures were performed.

### 2.3. Surgical Preparation

At the exact onset of their designated postmortem intervals, a midline abdominal incision was made to access the abdominal cavity. Prior to experimental perfusion, all harvested kidneys were subjected to a gross morphological examination by the surgical team.

The renal artery and ureter were separately cannulated using 26G intravenous polyurethane cannulas (Beybi Plastik, Istanbul, Turkey). The renal vein was severed near the hilus to perform nephrectomy, and the kidneys were weighed before being placed in a container for perfusion.

The total procedure time, from the start of the surgical incision to successful cannulation and initiation of perfusion, ranged from 5 to 10 min per kidney.

A kidney was excluded from the study if it met either of the following conditions: (1) the presence of macroscopic anatomical abnormalities (e.g., cysts, signs of previous trauma, or major vascular variations) observed during the initial surgical examination; or (2) the complete failure to produce any urine (anuria) despite completing the minimum 30 min perfusion protocol, which indicated an intrinsic or technical failure to establish a functional renal circuit.

### 2.4. Establishment of the Isolated Kidney Perfusion System

The perfusion solution was prepared by adding inulin (Alfa Aesar, Haverhill, MA, USA) 1000 mg/L, creatinine (Santa Cruz Biotechnology, Inc., Dallas, TX, USA) 1.3 mg/dL, sodium (Tekkim Kimya, Istanbul, Turkey) 140 mEq/L, potassium (Tekkim Kimya, Istanbul, Turkey) 5 mEq/L, and chloride (Tekkim Kimya, Istanbul, Turkey) 145 mEq/L to distilled water (BRK Kimya, Izmir, Turkey). The primary target for the perfusate was to achieve an osmolality suitable for tissues (~290 mOsm/L) [18,19,20]. However, the perfusate was not pH-buffered and was delivered at room temperature without active oxygenation to avoid any artificial interference with the natural progression of postmortem decay.

**Figure 1:**
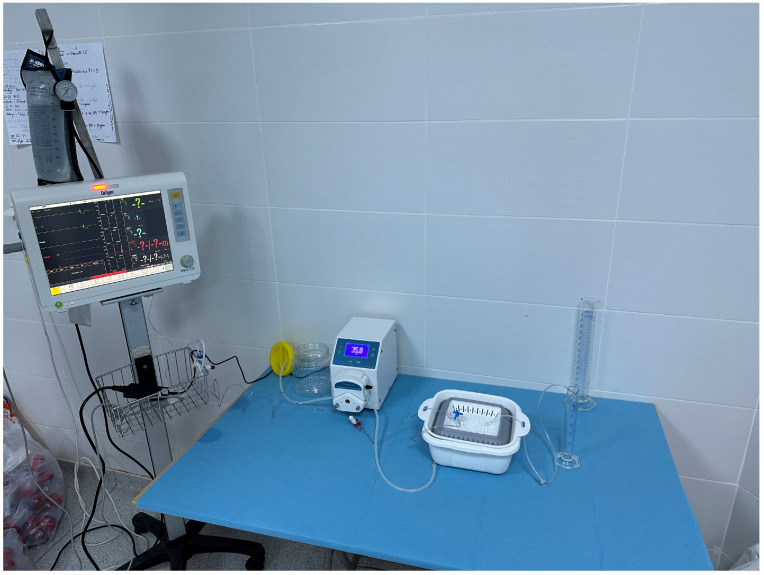
Perfusion system.

**Figure 2:**
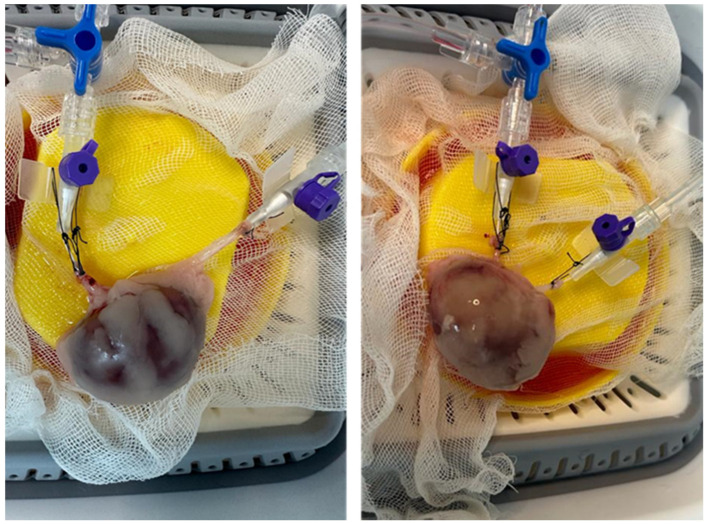
Kidney at the beginning of perfusion (**left**) and in the advanced stage of perfusion (**right**).

The isolated kidney perfusion was established as an open-loop (non-recirculating) system (Figure 1). One end of the silicone tubing of the peristaltic pump (Bimetron PSB-100) (Bimetron, Izmir, Turkey) was connected to the container holding the perfusion solution, and the other end was attached to a three-way stopcock connecting the pressure transducer (Metko Medikal, Ankara, Turkey) and the renal artery cannula. The pressure transducer was also connected to a patient monitor (Dräger Vista 120) (Dräger, Lübeck, Germany). The pressure transducer was calibrated before each perfusion run according to the manufacturer’s instructions. A total of 60 L of perfusion solution was prepared for the entire experimental study to ensure consistent biochemical composition across all groups. Because the isolated kidney perfusion was designed as an open-loop (non-recirculating) system, the exact volume of perfusate consumed by each individual kidney was not actively measured. Instead, an ample and continuous flow was driven by the peristaltic pump to strictly maintain the target arterial pressure (80–90 mmHg) throughout the procedure (Figure 2). The perfusion procedure was planned to continue for a minimum duration of 30 min, or until a target urine volume of 1 mL was successfully collected.

### 2.5. Biochemical Analyses and Evaluation

To mitigate any potential observer bias due to the lack of investigator blinding during the experimental phase, the functional outputs were measured objectively using automated devices. The anthrone method [21] was used for the measurement of inulin in urine employing a PerkinElmer VICTOR Nivo Multimode Plate Reader (PerkinElmer, Waltham, MA, USA). Creatinine measurement in urine was conducted using the “enzymatic method,” while potassium, sodium, and chloride were measured using the Ion Selective Electrode method; all these procedures were utilized through the Beckman Coulter AU5800 (Beckman Coulter Inc., Brea, CA, USA) device. Each sample was measured three times, and the average of these three measurements was used for statistical evaluation.

### 2.6. Calculations

The following calculations were made using the amounts of urine obtained during the experiment, the duration of perfusion (considered as the urine collection period), the concentrations of substances in the prepared perfusion solution, and the concentrations of substances in urine obtained from biochemical analyses:

- Urine Flow Rate = (urine volume)/(urine collection time);
- Renal Clearance = (urine concentration of the substance × urine flow rate)/(perfusion solution concentration of the substance);
- Filtered Load = Glomerular filtration rate (GFR) × perfusion solution concentration of the substance;
- Excreted Load = urine flow rate × urine concentration of the substance;
- Reabsorbed Load = filtered load − excreted load.

It was assumed that the GFR is equal to inulin clearance [22]. Urine flow, inulin clearance/GFR, and the values for filtered, excreted, and reabsorbed loads of substances were adjusted based on kidney weight.

### 2.7. Statistical Analysis

Data collected within the scope of the study were analyzed using the IBM Statistical Package for the Social Sciences (SPSS) for MacOS 29.0 (IBM Corp., Armonk, NY, USA). To prevent the risk of pseudoreplication arising from the physiological and temporal dependence of two kidneys derived from the same subject, the statistical unit of analysis was defined as the individual rabbit (*n* = 16). Prior to aggregating the bilateral data at the subject level to eliminate pseudoreplication bias, preliminary paired evaluations revealed no statistically significant functional differences between the right and left kidneys (*p* > 0.05). Functional data from the right and left kidneys of each rabbit were mathematically averaged to obtain a single representative value per subject. In instances where a single kidney was excluded due to anuria, the functional values from the successfully perfused contralateral kidney of that subject were utilized. Consequently, the final statistical evaluation included exactly 16 independent subjects, evenly distributed among the four categorical postmortem interval groups (*n* = 4 per group). Descriptive statistics are presented as mean ± standard deviation (SD), and median (minimum-maximum) values. The assumptions of normality were evaluated using the Shapiro–Wilk test. The consistency between repeated laboratory measurements was evaluated using Intraclass Correlation Analysis. Because the continuous variables were non-normally distributed and the sample size per postmortem interval group was small (*n* = 4), comparisons among the four groups were performed using the non-parametric Kruskal–Wallis H-test. Due to the categorical nature of the predetermined postmortem intervals and the lack of significant variance among groups, subsequent correlation or regression analyses were not deemed statistically applicable. Subgroup comparisons based on sex were evaluated using the Mann–Whitney U test. To mitigate observer bias, all biochemical measurements and subsequent statistical analyses were performed blinded to the postmortem interval group allocations. Results with a *p*-value of less than 0.05 were considered statistically significant. The complete dataset and statistical analysis outputs are provided as Supplementary Files S2 and S3.

## 3. Results

During the experimental procedure, 30 of the 32 initially perfused kidneys successfully produced urine within the planned parameters. Based on the exclusion criteria, the two anuric kidneys (one in the PM-5 group and one in the PM-15 group) were excluded. To completely eliminate pseudoreplication bias, the functional outputs were aggregated at the subject level by averaging the data from the right and left kidneys. For the two rabbits with a single functioning kidney, the data from that functioning kidney were utilized. As a result, the statistical analyses were robustly conducted on exactly 16 independent subjects (PM-1: *n* = 4; PM-5: *n* = 4; PM-10: *n* = 4; PM-15: *n* = 4).

To ensure that the observed functional outcomes were not confounded by sex differences, subgroup evaluations were performed. Statistical analyses (Mann–Whitney U test) revealed no significant sex-based differences across any of the measured functional metrics, including adjusted urine flow and inulin clearance (*p* > 0.05 for all metrics).

The Intraclass Correlation Analysis results, which evaluated the consistency between repeated measurements of sodium, potassium, chloride, creatinine, and inulin concentrations in urine, are presented in Table 1. The table shows that all parameters were statistically significant and consistent.

The distribution of kidney weights and the primary functional finding, adjusted urine flow, are presented in Table 2. Accordingly, adjusted urine flow rates were 0.0033 ± 0.0009 mL/min/g in the PM-1 group, 0.0034 ± 0.0009 mL/min/g in the PM-5 group, 0.0086 ± 0.0031 mL/min/g in the PM-10 group, and 0.0044 ± 0.0025 mL/min/g in the PM-15 group. Consistent with the rabbit-level analysis, no statistically significant differences were found among the groups overall (*p* = 0.069).

The inulin concentration in urine and the calculated adjusted inulin clearance, filtered inulin load, and excreted inulin load are detailed in Table 3. Following the statistical adjustment to correct for pseudoreplication, none of the inulin handling parameters, including urinary concentration (*p* = 0.698), adjusted clearance (*p* = 0.056), filtered load (*p* = 0.056), and excreted load (*p* = 0.059), exhibited any statistically significant chronological differences among the postmortem interval groups.

The results for potassium concentration in urine, along with adjusted potassium clearance, filtered potassium load, excreted potassium load, and reabsorbed potassium load, are shown in Table 4. Urinary potassium concentration did not significantly differ among the groups (*p* = 0.907). Furthermore, all functional load parameters failed to show statistically significant variations across the chronological milestones (all *p* > 0.05). Notably, the calculated reabsorbed potassium load yielded negative values across all groups. While in a living physiological state negative values indicate active tubular secretion, in this advanced postmortem ischemia model, it strongly suggests a massive passive leakage of intracellular potassium into the tubular fluid due to cellular autolysis.

The sodium concentration in urine and related metrics are described in Table 5. Despite apparent descriptive numerical variations among the groups, the Kruskal–Wallis test revealed that adjusted sodium clearance (*p* = 0.069), filtered sodium load (*p* = 0.056), excreted sodium load (*p* = 0.093), and reabsorbed sodium load (*p* = 0.846) did not statistically differ across the postmortem intervals. Similar to potassium, calculated sodium reabsorption yielded primarily negative values, indicating the complete absence of active physiological tubular reabsorption.

Table 6 details the analytical outcomes for chloride parameters. In parallel with sodium dynamics, none of the chloride handling parameters, including clearance, filtered load, and excreted load, demonstrated any statistically significant overall differentiation across the studied groups (all *p* > 0.05).

The measurement of creatinine concentrations in urine and the calculated values of adjusted creatinine clearance, filtered creatinine load, and excreted creatinine load are shown in Table 7. Urine creatinine concentrations were 2.5 ± 1.5 mg/dL in PM-1, 1.5 ± 0.6 mg/dL in PM-5, 1.0 ± 0.2 mg/dL in PM-10, and 1.3 ± 0.3 mg/dL in PM-15. As with all other evaluated solutes, urine creatinine parameters did not exhibit any statistically significant overall difference among the postmortem intervals (all *p* > 0.05).

## 4. Discussion

The estimation of the postmortem interval (PMI) is a fundamental challenge in forensic medicine. While macroscopic, biochemical, and molecular methods are well-documented [4,6,14,23], organ-level functional approaches remain largely underexplored [2]. This pilot study investigated the feasibility of utilizing an isolated ex vivo kidney perfusion model to assess residual postmortem renal function—specifically glomerular filtration and tubular solute handling—as a potential chronological marker for PMI.

The experimental postmortem intervals (1, 5, 10, and 15 h) were not chosen arbitrarily; they were specifically selected based on the known differential metabolic activities and hypoxia resistance of nephron segments. Previous histopathological and enzyme histochemical studies have demonstrated a sequential progression of postmortem changes in the kidney [13]. Due to high baseline Na,K-ATPase activity, the distal tubules and the thick ascending limb of Henle’s loop undergo severe autolysis between 5 and 10 h postmortem, whereas the proximal tubules exhibit prominent morphological changes, such as cytoplasmic vacuolization, around 15 h [13,14,24]. We originally hypothesized that this sequential structural degradation would functionally manifest as measurable, time-dependent alterations in specific solute clearances—for instance, an early failure in sodium, chloride, and potassium handling, followed by a later failure in water reabsorption leading to increased urine flow.

However, our experimental results did not support this anticipated physiological degradation pattern. Following statistical analysis none of the measured functional parameters—including adjusted urine flow, inulin clearance, and solute loads—exhibited statistically significant chronological variation across the evaluated postmortem intervals.

The primary functional indicator, adjusted inulin clearance (serving as a proxy for GFR), was markedly depressed, ranging between 0.0031 and 0.0086 mL/min/g across the groups. In the literature, in vivo rabbit inulin clearance is reported at approximately 0.43 ± 0.06 mL/min/g [9], while ex vivo models using autologous blood or optimized colloids report GFRs ranging from 0.217 to 0.787 mL/min/g [25,26,27]. When comparing our results to these studies, a distinct limitation of comparability must be acknowledged. Previous ex vivo studies were primarily designed for organ preservation, thus utilizing actively oxygenated, normothermic, and pH-buffered perfusion solutions to resuscitate and maintain cellular viability. In contrast, our study intentionally employed an unoxygenated, unbuffered, room-temperature crystalloid perfusate to mimic the natural progression of postmortem decay without artificially reviving the tissue. Under these specific, unoptimized forensic conditions, functional pressure-driven glomerular filtration was virtually nonexistent.

Tubular function outcomes further corroborated the absence of active physiological capacity. In a living kidney, the reabsorption and secretion of solutes rely on highly energy-dependent active transport mechanisms, notably the basolateral Na,K-ATPase pumps [28,29]. In our study, the filtered and excreted loads of the evaluated solutes were nearly identical, indicating negligible active reabsorption. Furthermore, the calculated reabsorbed loads for potassium, and to a large extent sodium and chloride, yielded predominantly negative values. While negative reabsorption values in a living physiological state denote active tubular secretion, in the context of advanced postmortem warm ischemia and rapid intracellular ATP depletion, active secretion is highly improbable. Instead, as originally considered in our methodological rationale, this phenomenon—particularly the consistently higher potassium concentration in the urine compared to the perfusate—is most likely attributable to a massive, passive intracellular leakage. As the selective permeability of the cell membrane is lost due to anoxia and subsequent autolysis, intracellular electrolytes passively diffuse into the necrotic tubular fluid [13].

In forensic science, the concept of “supravital reactions” refers to the period during which tissues can survive and respond to stimuli under ischemic conditions [1]. For instance, skeletal muscle is known to maintain electrical and mechanical excitability for up to 20 h postmortem [1,3]. We hypothesized that a similar prolonged supravital functional period might exist for the kidneys. However, our findings indirectly suggest that the highly metabolically active renal tissue lacks the functional resilience of skeletal muscle. Due to its substantial basal energy demands and profound vulnerability to hypoxia, the complex functional architecture of the kidney ceases to operate efficiently almost immediately following somatic death and the cessation of circulation.

### Limitations of the Study

Several limitations in our experimental design must be acknowledged, providing critical context for these results and guiding future methodological exploration.

First, the omission of the 0–1 h postmortem interval represents a major limitation. Renal tissue is highly sensitive to warm ischemia [11,12], and the severe functional depression observed at our earliest time point (1 h) indicates that meaningful residual renal function was likely lost prior to this period. Testing earlier intervals (e.g., minutes to a few hours) might have been significantly more informative for assessing residual functional capacity.

Second, the deliberate omission of active oxygenation, normothermia, and physiological pH buffering in the perfusion model—aimed at simulating a natural postmortem environment—undoubtedly accelerated cellular death and limited the demonstration of useful renal function. Highly metabolically active renal tissue is profoundly vulnerable to ischemic injury [11], and the use of a non-oxygenated, non-buffered, room-temperature crystalloid perfusion system without optimized colloidal support likely heavily impairs microcirculatory perfusion [25], preventing any meaningful preservation of renal physiological function. Therefore, the observed absence of functional activity may reflect the limitations of the experimental setup itself rather than exclusively a true postmortem physiological decline. These fundamental limits affect the biological interpretability of our findings and must be explicitly acknowledged.

Third, the use of high-dose CO_2_ for euthanasia may have introduced a pre-analytical bias. CO_2_ asphyxiation can induce acute physiological changes, including respiratory acidosis and intracellular electrolyte shifts (such as potassium alterations). Although these acute changes were likely overshadowed by the subsequent prolonged postmortem global hypoxia, their potential impact on the baseline biochemical parameters cannot be entirely ruled out.

Fourth, a limitation of this pilot study is the omission of parallel histopathological analyses, which precluded the direct correlation of the observed functional failures with the exact microscopic extent of cellular autolysis.

Finally, the proposed ex vivo isolated organ perfusion is a technically complex and resource-intensive laboratory procedure. While this study was designed as a methodological exploration, the practical applicability of such techniques in routine forensic medico-legal casework is highly limited.

## 5. Conclusions

This pilot study explored the feasibility of utilizing an isolated kidney perfusion model to measure residual organ-level functional capacity for postmortem interval estimation. Within this specific experimental setup—utilizing an unoxygenated, room-temperature crystalloid perfusion—the model failed to demonstrate reproducible renal function useful for PMI estimation. The results did not support the hypothesis that the prolonged supravital physiology observed in skeletal muscle can be directly extrapolated to the kidney. Postmortem renal tissue behaved essentially as a passive conduit, characterized by negligible active reabsorption and the passive leakage of intracellular electrolytes, with no statistically significant chronological differentiation between 1 and 15 h.

Future preliminary studies aiming to explore supravital renal function should strongly consider shifting their focus to the immediate early postmortem period (e.g., 0–1 h). Furthermore, integrating advanced methods from organ preservation research—such as actively oxygenated, temperature-controlled, and pH-buffered perfusates—may be necessary to unmask residual cellular functions. However, researchers must carefully balance the use of such artificial physiological support with the objective of reflecting the natural progression of postmortem decay. Ultimately, under these conditions, the absence of measurable renal function should be interpreted with caution, as it may reflect limitations of the experimental perfusion model, particularly impaired microcirculatory perfusion, rather than exclusively true postmortem physiological decline.

## Figures and Tables

**Table 1:** Results of Intraclass Correlation Analysis evaluating the agreement between repeated measurements of substance concentrations in urine.

Substances	Intraclass Correlation (Cronbach’s Alpha)	95% Confidence Interval	*p*-Value
Inulin concentration	0.781	0.599–0.889	<0.001
Sodium concentration	1.000	1.000–1.000	<0.001
Potassium concentration	1.000	1.000–1.000	<0.001
Chloride concentration	1.000	0.999–1.000	<0.001
Creatinine concentration	1.000	0.999–1.000	<0.001

**Table 2:** Distribution of the kidney weights and adjusted urine flow according to the experimental groups.

Variables		PM-1 Group (*n* = 4)	PM-5 Group (*n* = 4)	PM-10 Group (*n* = 4)	PM-15 Group (*n* = 4)	*p*-Value
Kidney weight (g)	Mean ± SD	20.5 ± 2.0	16.5 ± 1.3	16.5 ± 1.9	20.8 ± 3.8	0.054
	Median (Min–Max)	20.5 (18.0–23.0)	16.5 (15.0–18.0)	16.0 (15.0–19.0)	21.0 (17.0–24.0)	
Adjusted urine flow (ml/min/g)	Mean ± SD	0.0033 ± 0.0009	0.0034 ± 0.0009	0.0086 ± 0.0031	0.0044 ± 0.0025	0.069
	Median (Min–Max)	0.0030 (0.0026–0.0046)	0.0035 (0.0025–0.0043)	0.0093 (0.0044–0.0114)	0.0048 (0.0014–0.0066)	

**Table 3:** Distribution of urinary inulin concentration, adjusted inulin clearance, filtered inulin load, and excreted inulin load by groups.

Variables		PM-1 Group (*n* = 4)	PM-5 Group (*n* = 4)	PM-10 Group (*n* = 4)	PM-15 Group (*n* = 4)	*p*-Value
Urine inulin concentration (mg/L)	Mean ± SD	951.3 ± 93.3	923.6 ± 79.3	928.7 ± 60.7	893.7 ± 55.2	0.698
	Median (Min–Max)	936.0 (854.4–1078.8)	914.1 (836.9–1029.2)	947.6 (842.7–976.8)	896.6 (833.9–947.6)	
Adjusted inulin clearance (ml/min/g)	Mean ± SD	0.0031 ± 0.0006	0.0032 ± 0.0006	0.0078 ± 0.0025	0.0041 ± 0.0025	0.056
	Median (Min–Max)	0.0030 (0.0027–0.0039)	0.0031 (0.0026–0.0038)	0.0086 (0.0042–0.0099)	0.0045 (0.0012–0.0063)	
Filtered inulin load (μg/min/g)	Mean ± SD	3.127 ± 0.555	3.156 ± 0.623	7.811 ± 2.527	4.094 ± 2.484	0.056
	Median (Min–Max)	2.946 (2.678–3.936)	3.132 (2.611–3.750)	8.625 (4.165–9.830)	4.469 (1.208–6.229)	
Excreted inulin load (μg/min/g)	Mean ± SD	3.094 ± 0.613	3.207 ± 0.672	7.802 ± 2.597	4.130 ± 2.618	0.059
	Median (Min–Max)	2.953 (2.512–3.959)	3.245 (2.543–3.794)	8.759 (4.016–9.676)	4.543 (1.042–6.390)	

**Table 4:** Distribution of urinary potassium concentration, adjusted potassium clearance, filtered, excreted, and reabsorbed potassium load by groups.

Variables		PM-1 Group (*n* = 4)	PM-5 Group (*n* = 4)	PM-10 Group (*n* = 4)	PM-15 Group (*n* = 4)	*p*-Value
Urine K concentration (mEq/L)	Mean ± SD	8.5 ± 2.8	8.9 ± 3.8	7.4 ± 1.0	9.8 ± 3.1	0.907
	Median (Min–Max)	8.3 (5.4–12.1)	8.1 (5.1–14.1)	7.7 (5.9–8.2)	9.6 (7.1–12.9)	
Adjusted K clearance (ml/min/g)	Mean ± SD	0.0053 ± 0.0014	0.0061 ± 0.0036	0.0117 ± 0.0032	0.0073 ± 0.0025	0.111
	Median (Min–Max)	0.0049 (0.0040–0.0073)	0.0046 (0.0038–0.0115)	0.0131 (0.0068–0.0136)	0.0079 (0.0037–0.0095)	
Filtered K load (μEq/min/g)	Mean ± SD	0.016 ± 0.003	0.016 ± 0.003	0.039 ± 0.013	0.021 ± 0.012	0.056
	Median (Min–Max)	0.015 (0.013–0.020)	0.016 (0.013–0.019)	0.043 (0.021–0.049)	0.022 (0.006–0.031)	
Excreted K load (μEq/min/g)	Mean ± SD	0.026 ± 0.007	0.031 ± 0.018	0.058 ± 0.017	0.036 ± 0.014	0.111
	Median (Min–Max)	0.024 (0.020–0.036)	0.023 (0.020–0.058)	0.065 (0.033–0.069)	0.040 (0.016–0.049)	
Reabsorbed K load (μEq/min/g)	Mean ± SD	−0.010 ± 0.008	−0.015 ± 0.016	−0.019 ± 0.007	−0.016 ± 0.007	0.337
	Median (Min–Max)	−0.007 (−0.022 to −0.005)	−0.009 (−0.039 to −0.004)	−0.018 (−0.028 to −0.012)	−0.014 (−0.025 to −0.010)	

**Table 5:** Distribution of urinary sodium concentration, adjusted sodium clearance, filtered, excreted, and reabsorbed sodium load according to groups.

Variables		PM-1 Group (*n* = 4)	PM-5 Group (*n* = 4)	PM-10 Group (*n* = 4)	PM-15 Group (*n* = 4)	*p*-Value
Urine Na concentration (mEq/L)	Mean ± SD	148.3 ± 16.2	140.2 ± 16.3	133.9 ± 8.4	137.0 ± 8.8	0.385
	Median (Min–Max)	144.3 (134.5–170.2)	145.2 (116.7–154.0)	133.2 (124.3–144.8)	140.0 (124.2–143.7)	
Adjusted Na clearance (ml/min/g)	Mean ± SD	0.0035 ± 0.0010	0.0034 ± 0.0008	0.0079 ± 0.0024	0.0042 ± 0.0023	0.069
	Median (Min–Max)	0.0031 (0.0028–0.0050)	0.0033 (0.0026–0.0045)	0.0086 (0.0046–0.0098)	0.0043 (0.0014–0.0066)	
Filtered Na load (μEq/min/g)	Mean ± SD	0.44 ± 0.08	0.44 ± 0.09	1.09 ± 0.35	0.57 ± 0.35	0.056
	Median (Min–Max)	0.41 (0.37–0.55)	0.44 (0.37–0.53)	1.21 (0.58–1.38)	0.63 (0.17–0.87)	
Excreted Na load (μEq/min/g)	Mean ± SD	0.48 ± 0.15	0.49 ± 0.12	1.10 ± 0.34	0.59 ± 0.34	0.093
	Median (Min–Max)	0.43 (0.37–0.70)	0.47 (0.38–0.63)	1.20 (0.62–1.39)	0.62 (0.17–0.95)	
Reabsorbed Na load (μEq/min/g)	Mean ± SD	−0.045 ± 0.069	−0.047 ± 0.039	−0.010 ± 0.093	−0.015 ± 0.087	0.846
	Median (Min–Max)	−0.021 (−0.145 to 0.009)	−0.035 (−0.104 to −0.015)	−0.011 (−0.119 to 0.103)	−0.041 (−0.084 to 0.104)	

**Table 6:** Distribution of urinary chloride concentration, adjusted chloride clearance, filtered, excreted, and reabsorbed chloride load by groups.

Variables		PM-1 Group (*n* = 4)	PM-5 Group (*n* = 4)	PM-10 Group (*n* = 4)	PM-15 Group (*n* = 4)	*p*-Value
Urine Cl concentration (mEq/L)	Mean ± SD	150.5 ± 17.2	141.0 ± 16.0	135.3 ± 8.5	137.7 ± 8.8	0.431
	Median (Min–Max)	146.2 (135.5–174.0)	146.5 (117.5–153.5)	135.0 (125.2–146.0)	140.6 (125.0–144.7)	
Adjusted Cl clearance (ml/min/g)	Mean ± SD	0.0034 ± 0.0010	0.0033 ± 0.0008	0.0077 ± 0.0023	0.0040 ± 0.0022	0.069
	Median (Min–Max)	0.0030 (0.0027–0.0048)	0.0032 (0.0026–0.0043)	0.0084 (0.0044–0.0095)	0.0042 (0.0014–0.0065)	
Filtered Cl load (μEq/min/g)	Mean ± SD	0.45 ± 0.08	0.46 ± 0.09	1.13 ± 0.37	0.59 ± 0.36	0.056
	Median (Min–Max)	0.43 (0.39–0.57)	0.45 (0.38–0.54)	1.25 (0.60–1.43)	0.65 (0.18–0.90)	
Excreted Cl load (μEq/min/g)	Mean ± SD	0.49 ± 0.15	0.49 ± 0.11	1.11 ± 0.34	0.59 ± 0.34	0.093
	Median (Min–Max)	0.44 (0.37–0.70)	0.48 (0.39–0.63)	1.22 (0.62–1.40)	0.62 (0.17–0.96)	
Reabsorbed Cl load (μEq/min/g)	Mean ± SD	−0.036 ± 0.067	−0.034 ± 0.034	0.018 ± 0.094	0.001 ± 0.092	0.760
	Median (Min–Max)	−0.013 (−0.132 to 0.015)	−0.022 (−0.083 to −0.007)	0.009 (−0.083 to 0.138)	−0.026 (−0.073 to 0.131)	

**Table 7:** Distribution of urine creatinine concentration, adjusted creatinine clearance, filtered, and excreted creatinine load according to groups.

Variables		PM-1 Group (*n* = 4)	PM-5 Group (*n* = 4)	PM-10 Group (*n* = 4)	PM-15 Group (*n* = 4)	*p*-Value
Urine Creatinine concentration (mg/dL)	Mean ± SD	2.5 ± 1.5	1.5 ± 0.6	1.0 ± 0.2	1.3 ± 0.3	0.142
	Median (Min–Max)	2.3 (1.1–4.4)	1.5 (0.8–2.3)	1.0 (0.8–1.2)	1.3 (1.0–1.6)	
Adjusted Creatinine clearance (ml/min/g)	Mean ± SD	0.0056 ± 0.0028	0.0040 ± 0.0022	0.0058 ± 0.0011	0.0038 ± 0.0015	0.272
	Median (Min–Max)	0.0046 (0.0038–0.0096)	0.0032 (0.0024–0.0073)	0.0061 (0.0042–0.0066)	0.0040 (0.0017–0.0054)	
Filtered Creatinine load (μg/min/g)	Mean ± SD	0.041 ± 0.007	0.041 ± 0.008	0.102 ± 0.033	0.053 ± 0.032	0.056
	Median (Min–Max)	0.038 (0.035–0.051)	0.041 (0.034–0.049)	0.112 (0.054–0.128)	0.058 (0.016–0.081)	
Excreted Creatinine load (μg/min/g)	Mean ± SD	0.071 ± 0.033	0.053 ± 0.029	0.075 ± 0.016	0.049 ± 0.022	0.382
	Median (Min–Max)	0.058 (0.048–0.118)	0.041 (0.033–0.095)	0.079 (0.053–0.088)	0.053 (0.020–0.071)	

## Data Availability

The original contributions presented in this study are included in the Supplementary Materials. Further inquiries can be directed to the corresponding author.

## Supplementary Materials

The following supporting information can be downloaded at: https://www.mdpi.com/article/10.3390/diagnostics16091266/s1, File S1: ARRIVE Guidelines 2.0 Author Checklist; File S2: Original Statistical Analysis (SPSS) Dataset; File S3: Original Statistical Analysis Output; File S4: Animal Experiments Local Ethics Committee Approval Document.

## Abbreviations

The following abbreviations are used in this manuscript:

PM	Postmortem
PMI	Postmortem interval
GFR	Glomerular filtration rate
SPSS	Statistical Package for the Social Sciences
