# Supplementary material for: Feasibility of an Isolated Kidney Perfusion Model for Postmortem Interval Estimation in a Rabbit Model: A Pilot Study
Source: Diagnostics (Basel). 2026 Apr 23;16(9):1266. doi: 10.3390/diagnostics16091266 (PMC13163118; doi:10.3390/diagnostics16091266)
Supplement: Supplementary file 1 [file diagnostics-16-01266-s001.zip › Supplementary File S4_EgeUniversity_AnimalEthicsApproval.pdf.pdf]

EGE ÜNİVERSİTESİ
HAYVAN DENEYLERİ YEREL ETİK KURULU

SAYI: 2021-066
KONU: Onay

28.07.2021

Etik kurulumuza yapmış olduğunuz başvuru doğrultusunda **"İZOLE EDILMIŞ BÖBREK PERFÜZYONU TEKNIĞININ POSTMORTEM İNTERVAL TAYININDE KULLANILABILIRLIĞI: TAVŞAN MODELI"** isimli araştırma projeniz değerlendirilmiştir.

Yürütücü: **Dr. Öğr. Üyesi Hülya GÜLER, EÜTF Adli Tıp AD**
Arş. Gör. Dr. Ramazan TEMÜRKOL, EÜTF Adli Tıp AD
Doç. Dr. Ahsen KAYA, EÜTF Adli Tıp AD
Prof. Dr. Yasemin AKÇAY, EÜTF Tıbbi Biyokimya AD
Doç. Dr. Ayşegül Keser, EÜTF Fizyoloji AD
Arş. Gör. Dr. Orhan Fahri DEMİR, EÜTF Plastik, Rekonstrüktif ve Estetik Cerrahi AD
Arş. Gör. Meltem KOCAMANOĞLU, EÜTF Tıbbi Biyokimya AD

Proje başvuru formunuzda belirtildiği koşullarda 27 adet Yeni Zelanda tavşanı deney hayvanı kullanarak araştırmayı gerçekleştirmeniz kurulumuz tarafından uygun bulunmuştur. Saygılarımla bilgilerinizi rica ederim.

Prof. Dr. N. Ülkü KARABAY YAVAŞOĞLU
(E.Ü. Hayvan Deneyleri Yerel Etik Kurulu Başkanı)

Prof. Dr. Altuğ YAVAŞOĞLU — Prof. Dr. Aytül ÖNAL

Prof. Dr. Hüseyin TEZEL — Prof. Dr. Figen KIRKPINAR

Prof. Dr. Haşmet ÇAĞIRGAN — Prof. Dr. Uğur KAYA

(KATILMADI)
Prof. Dr. Okan BİLGE — Doç. Dr. Tayfun YOLDAŞ

(KATILMADI)
Dr. Öğr. Üyesi Sumru SÖZER KARADAĞLI — Vet. Hek. F. Emrah SOYLU

(KATILMADI)
Özcan NALBANTOĞLU — Melek Merve AFŞAROĞLU
